# Sensorimotor network hypersynchrony as an endophenotype in families with genetic generalized epilepsy: A resting‐state functional magnetic resonance imaging study

**DOI:** 10.1111/epi.14663

**Published:** 2019-02-07

**Authors:** Chayanin Tangwiriyasakul, Suejen Perani, Eugenio Abela, David W. Carmichael, Mark P. Richardson

**Affiliations:** ^1^ Department of Basic and Clinical Neuroscience Institute of Psychiatry Psychology and Neuroscience King's College London London UK; ^2^ UCL Great Ormond Street Institute of Child Health, University College London London UK; ^3^ School of Imaging and Bioengineering Faculty of Life Sciences and Medicine King's College London London UK; ^4^ Centre for Epilepsy King's College Hospital London UK

**Keywords:** BOLD fMRI, endophenotype of GGE, genetic generalized epilepsy

## Abstract

Recent evidence suggests that three specific brain networks show state‐dependent levels of synchronization before, during, and after episodes of generalized spike‐wave discharges (GSW) in patients with genetic generalized epilepsy (GGE). Here, we investigate whether synchronization in these networks differs between patients with GGE (n = 13), their unaffected first‐degree relatives (n = 17), and healthy controls (n = 18). All subjects underwent two 10‐minute simultaneous electroencephalographic–functional magnetic resonance imaging (fMRI) recordings without GSW. Whole‐brain data were divided into 90 regions, and blood oxygen level–dependent (BOLD) phase synchrony in a 0.04–0.07‐Hz band was estimated between all pairs of regions. Three networks were defined: (1) the network with highest synchrony during GSW events, (2) a sensorimotor network, and (3) an occipital network. Average synchrony (mean node degree) was inferred across each network over time. Notably, synchrony was significantly higher in the sensorimotor network in patients and in unaffected relatives, compared to controls. There was a trend toward higher synchrony in the GSW network in patients and in unaffected relatives. There was no difference between groups for the occipital network. Our findings provide evidence that elevated fMRI BOLD synchrony in a sensorimotor network is a state‐independent endophenotype of GGE, present in patients in the absence of GSW, and present in unaffected relatives.

## 1 INTRODUCTION

There is strong evidence for the heritability of genetic (or idiopathic) generalized epilepsy (GGE).^1^ An important concept emerging in studies of disease inheritance is endophenotype,^2^ a heritable trait with a simpler genetic basis than the full disorder, which may be present in family members who do not have the disease. There is increasing interest in identifying endophenotypes in epilepsy.

A few studies have already demonstrated that GGE may have a distinct endophenotype. For example, we found enhanced electroencephalographic (EEG) network synchrony in patients with GGE and unaffected first‐degree relatives.^3^ Using functional magnetic resonance imaging (fMRI), hyperconnectivity between a network engaged in a cognitive task and the sensorimotor network was found in both patients with GGE and first‐degree relatives.^4^, ^5^ An endophenotype is a heritable trait that is a component of a disorder or associated with high liability to develop the disorder. An endophenotype may be present in family members who do not have the disease, hence increasing the power of genetic studies. This concept has allowed the genetic dissection of complex disorders such as rolandic epilepsy.^6^, ^7^

In a recent study, we found that synchrony in specific networks, observed with blood oxygen level–dependent (BOLD) fMRI and simultaneous EEG, varies dynamically around the time of generalized spike‐wave (GSW) events observed in EEG,^8^ apparently anticipating the onset of GSW by several seconds. We also noted that, remote from GSW events, there was evidence that network synchrony was higher in patients than healthy controls in a sensorimotor network. Here, we examine whether this elevated synchrony is also present in unaffected first‐degree relatives of patients with GGE.

## 2 MATERIALS AND METHODS

### 2.1 Participants

We have previously acquired and published EEG fMRI data from 21 patients diagnosed with juvenile myoclonic epilepsy (JME) or generalized tonic‐clonic seizures only (GTCSO)^8^; here, we include the 13 in whom fMRI runs were entirely free of GSW (mean age = 20.5 ± SD 6.6 years), in addition to 18 healthy controls reported in the same prior study (mean age = 23.9 ± SD 3.8 years), and 17 unaffected first‐degree relatives collected during the same period of time, but not previously reported (mean age = 39.4 ± SD 14.2 years; see Table S1). Note that none of the relatives was related to any of the patients in this study. Patients were recruited through clinics across southeast London. Relatives were recruited via patients with JME or GTCSO attending these clinics. Participants were excluded if they had any neurological diagnoses other than epilepsy or history of drug or alcohol misuse. Healthy controls and first‐degree relatives did not have any history of seizures or epilepsy. This study was approved by the Riverside Research Ethics Committee (REC approval number 12/LO/2006 and REC approval number 11/LO/1421), and all participants gave written informed consent according to the Declaration of Helsinki (2013).

### 2.2 Data acquisition and preprocessing

Participants underwent two runs of simultaneous resting‐state EEG fMRI on a 3T MR750 scanner (GE Healthcare) to acquire 300 BOLD echo‐planar images per run (3.3‐mm isotropic voxels, field of view = 211 mm, repetition time [TR] = 2.160 seconds, echo time = 25 milliseconds, flip angle = 75°, 36 slices, thickness = 2.5 mm). During scanning, all subjects were asked to rest with their eyes closed. EEG data were acquired at 5000 Hz with an MRI‐compatible EEG cap containing 63 Ag/AgCl electrodes referenced to FCz (Brain Products). Impedances were kept at <10 kOhm. Magnetic resonance gradient and pulse‐related artifacts were removed offline from the EEG recorded inside the MRI using template artifact subtraction (Brain Analyzer, Brain Products).^9^, ^10^ To preprocess fMRI data, we used SPM8 (r6313, www.fil.ion.ucl.ac.uk/spm) running on MATLAB (R2017b; MathWorks) and the FIACH package (www.homepages.ucl.ac.uk/~ucjttie/FIACH.html) to correct for physiological artifacts in the BOLD time series.^11^ Next, we normalized the corrected data into the standard Montreal Neurological Institute space. Finally, all images were spatially smoothed using a Gaussian kernel of 8 mm full width at half maximum.

### 2.3 fMRI data analysis

Full details of data analysis were reported in our previous study.^8^ In summary, BOLD signals were first bandpass filtered between 0.04 and 0.07 Hz.^12^ We then parcellated the brain into 90 regions using automatic anatomical labeling.^13^ The first principal component of voxel time series was used to represent each brain region.^14^ Next, the Hilbert transform was applied to estimate the instantaneous phase of the first principal component in each region. Subsequently, we estimated a time‐varying phase difference matrix by subtracting the phase angle between pairs of regions, resulting in a 90 × 90 × 285 adjacency matrix for each fMRI run. Note that for each run, the first 10 TRs and the last 5 TRs from 300 TRs were excluded because fMRI noise was seen in the EEG. We binarized these matrices using a threshold of pi/6.^8^, ^15^ Tensor decomposition was applied to the series of adjacency matrices for each run to try to reduce the number of spurious network connections.^8^

### 2.4 EEG data analysis

We used alpha power estimated from O1, O2, and Oz to monitor the level of vigilance of each subject in each run, to take account of likely change in vigilance over the duration of each scan run.^16^ To avoid fMRI noise in the EEG that would prevent estimation of alpha power, we excluded the first 21.6 seconds (10 TRs) and the last 10.8 second (5 TRs) of each EEG. Each EEG was bandpass filtered between 8 and 12 Hz. Then we estimated the alpha power over each consecutive period of 10 seconds. To avoid intersubject variability, we normalized the alpha power by dividing by the broadband EEG power (1‐40 Hz). For each run, we estimated the slope of normalized alpha power, representing a change in the level of vigilance. This slope was later used as a covariate.

### 2.5 Estimation of average network synchrony

In our previous study, we examined time‐varying network synchrony around the time of occurrence of GSW discharges, and around random events, in the same subjects.^8^ We observed three canonical networks in these data: (1) a network prominent during GSW in patients (GSW network), (2) a network prominent prior to GSW in patients (sensorimotor network), and (3) a network prominent in healthy controls at the time of random events (occipital network; see Table S2 for a list of brain regions included in each network). We estimated network synchrony in these three canonical networks. As in our previous study, we used mean degree to measure network synchrony.^8^ For each run, we took the phase synchrony matrices (90 × 90 × 285) obtained from the previous step and then subsampled by including only the regions within each network, resulting in matrices with *p *× *p *× 285 elements, where *p* is the number of regions in the network. At each TR, we estimated mean degree, which is the average of all elements in the *p *× *p* matrix. This step was repeated for each TR and averaged over all TRs in each run. Finally, we estimated normalized mean degree for each subject, which is the mean degree of each network divided by the mean degree over the entire brain (where *p* = 90).

### 2.6 Statistical analyses

Because the data in this study were nonnormally distributed, as determined by one‐sample Kolmogorov‐Smirnov test, nonparametric methods were chosen. We first ran a rank analysis of covariance (Quade test) to examine mean degree across the three groups,^17^ where age and level of vigilance were used as covariates. A Mann‐Whitney test was used to compare between pairs of groups. We considered the results to be significant if *P* < 0.05 after Bonferroni correction for three group comparisons.

## 3 RESULTS

**Figure 1:**
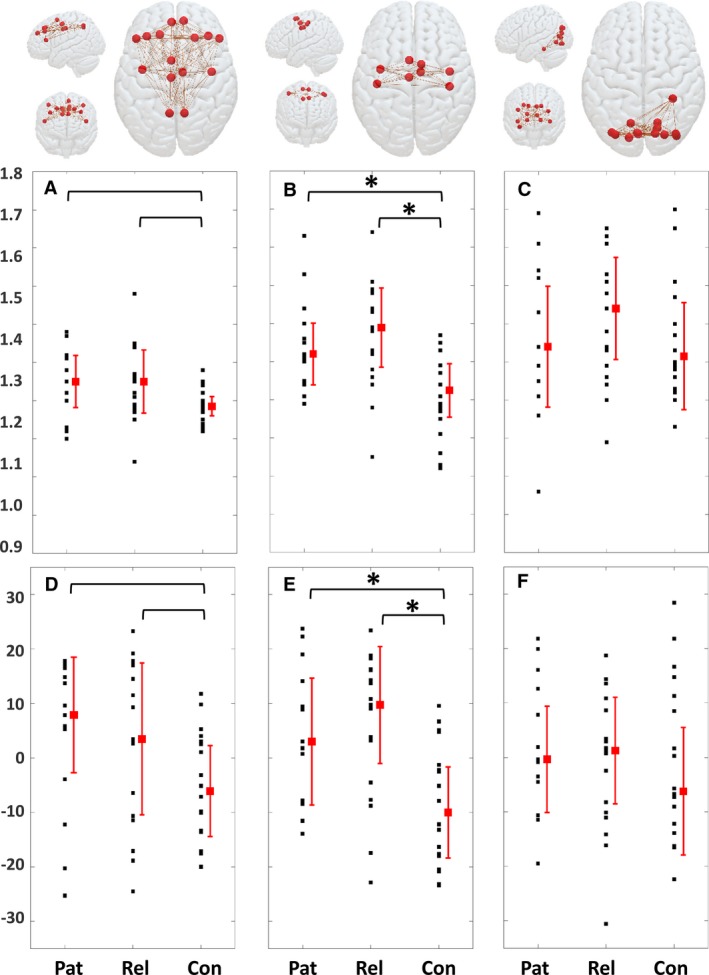
Average level of functional magnetic resonance imaging blood oxygen level–dependent phase synchrony in the three canonical networks. A, D, Generalized spike‐wave network; B, E, sensorimotor network; C, F, occipital network. The top row shows drawings of the networks involved. A‐C, Mean degree uncorrected for age and level of vigilance. D‐F, Mean degree rank (centered on zero) adjusted for age and level of vigilance using Quade analysis of variance. *Bracketed comparisons with probability values that survive Bonferroni correction at *P* < 0.05. Con, controls; Pat, patients; Rel, relatives

Patients and first‐degree relatives had significantly higher network synchrony (mean degree) in the sensorimotor network than in the control group, after adjustment for age and level of vigilance and Bonferroni correction (Figure 1 and Table 1). There was a nonsignificant trend toward higher network synchrony in patients and first‐degree relatives in the GSW network. There were no differences between groups in the occipital network.

**Table 1:** Probaility values for group comparisons of synchrony in each of the three canonical networks

	Network	Quade ANOVA	Mann‐Whitney *U*		
			Patients vs relatives	Patients vs controls	Relatives vs controls
Bonferroni corrected and adjusted for age and level of vigilance	GSW	0.105	1.000	0.102	0.225
	Sensorimotor	0.002^a^	1.000	0.024^a^	0.006^a^
	Occipital	0.776	1.000	1.000	1.000

We report here probability values with Bonferroni correction and adjustment for age and level of vigilance. ANOVA, analysis of variance; GSW, generalized spike‐wave.

a Significant (*P* < 0.05).

## 4 DISCUSSION

In this study, we found that mean degree, a measure reflecting the average level of BOLD signal phase synchronization, was significantly higher in a sensorimotor network in patients with GGE and in relatives of patients with GGE than in healthy control subjects. The data were obtained with simultaneous EEG and were free from episodes of GSW, suggesting that this phenomenon is independent of seizures or epilepsy, and may represent an inherited endophenotype of GGE. There was also a trend that mean degree was higher in the interictal state in patients, and in relatives, in the network that becomes prominently synchronized during GSW.

### 4.1 Network connectivity in GGE: State versus trait

In our previous study,^8^ we found that phase synchronization of BOLD signals in canonical brain networks varied over time, in particular showing differences in epochs around GSW events compared to epochs without. This finding suggests that brain network synchronization may vary over seconds or longer prior to GSW onset on EEG, and may reflect the mechanisms responsible for the transition from normal brain activity to GSW. In the study reported here, we found that brain network synchrony was abnormally elevated in GGE patients remote from GSW events as well as in first‐degree relatives, suggesting this phenomenon is an invariant trait. In support of this suggestion, several other studies using various data modalities, including diffusion tensor imaging, fMRI, and transcranial magnetic brain stimulation, have reported hyperconnectivity in sensorimotor‐related areas of patients with GGE.^4^, ^18^, ^19^

### 4.2 Network connectivity in relatives of patients with GGE: Endophenotype

In a previous study using EEG,^3^ we studied features of functional networks. We found, exclusively in the low‐alpha 6–9‐Hz range, that clustering coefficient and the variance of mean degree differed between GGE patients and healthy controls, and also differed between relatives of GGE patients and healthy controls. These measures were global statistics of a whole‐brain network, and we did not attempt to examine specific subnetworks, such as a sensorimotor network. In subsequent theoretical work, we showed that the connectivity features of these networks specifically predispose them to ictal onset.^20^

A previous study using fMRI in JME patients and their first‐degree relatives showed that connectivity between the network involved in a working memory task carried out during scanning, and a sensorimotor network, was increased both in patients and unaffected relatives.^5^ Our study here extends these findings to show that excessive synchrony within the sensorimotor network itself is observable at rest. Although the relationship between observation of sensorimotor network hypersynchrony and the mechanism of GSW onset cannot be inferred from our data, we speculate that the endophenotype of sensorimotor hypersynchronization plays a role in facilitating the engagement of large‐scale brain circuits in GSW driven from localized nodes such as the precuneus.^21^

### 4.3 Strengths and weaknesses of our study

Our findings of elevated synchronization in brain networks at rest, without GSW, could only be made because we had simultaneous EEG. For obvious reasons, it would be impossible to say that the network phenomena we observed are state‐independent unless we could exclude the occurrence of GSW events during the fMRI scans. Our subject groups differ in age distribution, but our robust methodology (nonparametric statistics with inclusion of age and level of vigilance as covariates) allowed us to take an optimal approach despite this limitation. Furthermore, in post hoc analyses using Mann‐Whitney *U* test, we showed that there was no effect in patients or relative groups of gender, of GGE syndrome (ie, effects were similar in male and female and in the JME and GTCSO groups), or of photosensitivity (see details in the Table S3).

### 4.4 Conclusion and future work

We found here evidence that fMRI BOLD hypersynchrony in a sensorimotor network is an endophenotype of GGE, present in patients and unaffected relatives. Future work should seek to understand the mechanisms and genetic underpinnings of this observation. The sensorimotor system is amenable to manipulation by techniques such as noninvasive brain stimulation (eg using transcranial magnetic stimulation). This might allow the clinical relevance of the sensorimotor hypersynchrony to be tested in the future.

## DISCLOSURE

The authors have no conflicts of interest to report. We confirm that we have read the Journal’s position on issues involved in ethical publication and affirm that this report is consistent with those guidelines.
