# Supplementary material for: Sensorimotor network hypersynchrony as an endophenotype in families with genetic generalized epilepsy: A resting‐state functional magnetic resonance imaging study
Source: Epilepsia. 2019 Feb 7;60(3):e14–9. doi: 10.1111/epi.14663 (PMC6446943; doi:10.1111/epi.14663)
Supplement: Supplementary file 1 [file EPI-60-e14-s001.docx]

**Appendix**

|  | | **GGE patients** | | | | **Unaffected GGE relatives** | | | **Healthy controls** | |
| --- | --- | --- | --- | --- | --- | --- | --- | --- | --- | --- |
| **Age** | **Gender** | | **Syndrome** | **PS** | **AEDs** | **Age** | **Gender** | **Syndrome** | **Age** | **Gender** |
| 16 | Female | | GTCSO | Y | None | 22 | Female | GTCSO | 17 | Female |
| 17 | Female | | GTCSO | N | None | 24 | Female | GTCSO | 20 | Female |
| 39 | Male | | GTCSO | N | LEV | 42 | Female | GTCSO | 20 | Female |
| 13 | Female | | JME | Y | None | 46 | Female | GTCSO | 22 | Female |
| 15 | Female | | JME | N | None | 52 | Female | GTCSO | 23 | Female |
| 16 | Female | | JME | Y | None | 25 | Male | GTCSO | 23 | Female |
| 20 | Female | | JME | N | None | 33 | Male | GTCSO | 25 | Female |
| 22 | Female | | JME | N | LMT, LEV | 51 | Male | GTCSO | 25 | Female |
| 22 | Female | | JME | N | LMT | 22 | Female | JME | 28 | Female |
| 26 | Female | | JME | N | None | 27 | Female | JME | 21 | Male |
| 20 | Male | | JME | -- | None | 37 | Female | JME | 22 | Male |
| 20 | Male | | JME | Y | VPA, LEV | 51 | Female | JME | 23 | Male |
| 21 | Male | | JME | Y | VPA | 55 | Female | JME | 24 | Male |
|  |  | |  |  |  | 58 | Female | JME | 24 | Male |
|  |  | |  |  |  | 18 | Male | JME | 24 | Male |
|  |  | |  |  |  | 52 | Male | JME | 26 | Male |
|  |  | |  |  |  | 56 | Male | JME | 29 | Male |
|  |  | |  |  |  |  |  |  | 34 | Male |

Table A1

Demographic characteristics of the three subject groups. Abbreviations: GGE, Genetic Generalised Epilepsy; JME, Juvenile Myoclonic Epilepsy; GTCSO, Generalized Tonic-Clonic Seizures Only (GCTSO); PS, Photosensitivity; Y, Yes; N, No; --, data is not available; LEV, Levetiracetam; LMT, Lamotrigine; VPA, Valproic acid

| **GSW** | **Sensorimotor** | **Occipital** |
| --- | --- | --- |
| 'Precentral_L' | 'Precentral_L' | 'Calcarine_L' |
| 'Precentral_R' | 'Precentral_R' | 'Calcarine_R' |
| 'Frontal_Sup_L' | 'Supp_Motor_Area_L' | 'Cuneus_L' |
| 'Frontal_Sup_R' | 'Supp_Motor_Area_R' | 'Cuneus_R' |
| 'Frontal_Mid_L' | 'Cingulum_Mid_L' | 'Lingual_L' |
| 'Frontal_Mid_R' | 'Cingulum_Mid_R' | 'Lingual_R' |
| 'Frontal_Inf_Tri_L' | 'Postcentral_L' | 'Occipital_Sup_L' |
| 'Frontal_Inf_Tri_R' | 'Postcentral_R' | 'Occipital_Sup_R' |
| 'Supp_Motor_Area_L' |  | 'Occipital_Mid_L' |
| 'Frontal_Sup_Medial_L' |  | 'Occipital_Mid_R' |
| 'Frontal_Sup_Medial_R' |  | 'Occipital_Inf_L' |
| 'Cingulum_Mid_L' |  | 'Occipital_Inf_R' |
| 'Cingulum_Mid_R' |  | 'Fusiform_R' |
| 'Precuneus_L' |  |  |
| 'Precuneus_R' |  |  |

Table A2

Three canonical networks (GSW, sensorimotor and occipital networks) and their nodes. Abbreviations: L/R, left/right; Sup, superior; Mid, middle; Inf, Inferior; Supp, supplemental^1^.

Reference

1. Tangwiriyasakul C, Perani S, Centeno M, et al. Dynamic brain network states in human generalized spike-wave discharges. *Brain* (October 2018).

| Network | Patients | | | Relatives | |
| --- | --- | --- | --- | --- | --- |
|  | By Sex | By Syndrome | By Photosensitivity | By Sex | By Syndrome |
| GSW | 0.64 | 0.24 | 0.08 | 0.76 | 0.44 |
| Sensorimotor | 1.00 | 0.40 | 0.81 | 0.92 | 0.50 |
| Occipital | 0.44 | 0.61 | 0.29 | 0.92 | 0.44 |

Table A3

P-values from Mann-Whitney U test for group comparisons in each of the three canonical network. We report here p-values after adjustment for age and level of vigilance.


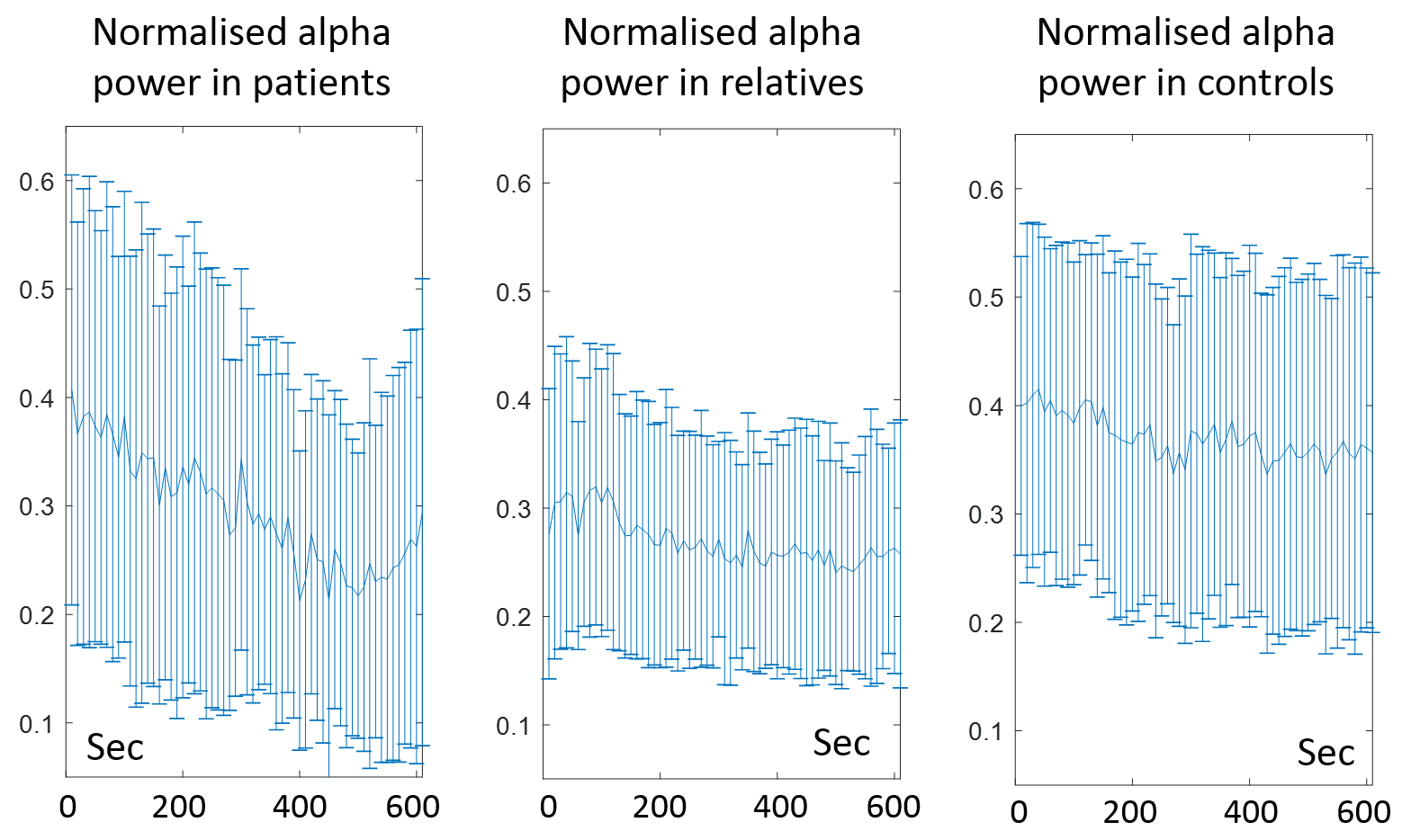


Figure A1: Evolution of averaged normalised alpha power and its standard deviation estimated across O1, O2, and Oz (from left to right: patients, 1^st^-degree relatives, and controls) over the course of time. Note that: we found no significant difference among the level of vigilance between the groups (slope of normalise alpha).
